# Age-related results over 2 years of the multicenter Spanish study of atropine 0.01% in childhood myopia progression

**DOI:** 10.1038/s41598-023-43569-x

**Published:** 2023-09-28

**Authors:** Inés Pérez-Flores, Beatriz Macías-Murelaga, Multicenter Group of Atropine Treatment for Myopia Control (GTAM), Inés Pérez Flores, Marta Valcárcel Vizcaíno, Marta García Arias, Sara Catalán López, Manuel Rodríguez Enríquez, María Iglesias Álvarez, Betty Lorente Bulnes, Matías García-Anllo Reinoso, José María Carnero, Victoria de Rojas Silva, Jesús Barrio Barrio, David Rodríguez Feijoo, Javier Rodríguez Sánchez, Argentina Rosario Calvo Robles, Sonia López-Romero Moraleda, Ángela Barrajón Rodríguez, Javier Gálvez Martínez, Diana Victoria Mesa Carina, Elena Galán Risueño, Esther Rodríguez Domingo, Jesús Barrio-Barrio

**Affiliations:** 1 Department of Ophthalmology, Ribera Povisa Hospital, Vigo, Spain; 2 Department of Ophthalmology, Cruces University Hospital, Barakaldo, Spain; 3 Department of Ophthalmology, Navarra University Clinic Hospital, Navarra Institute for Health Research, IdiSNA, Pio XII, 36. Pamplona, 31008 Navarra, Spain; 4 Department of Ophthalmology, Vigo University Hospital, Vigo, Spain; 5 Department of Ophthalmology, Lugo University Hospital, Lugo, Spain; 6 Department of Ophthalmology, Ourense University Hospital, Ourense, Spain; 7 Lorente Clinic, Ourense, Spain; 8 Department of Ophthalmology, A Coruña University Hospital, A Coruña, Spain; 9 Victoria de Rojas Ophthalmologic Institute, A Coruña, Spain; 10 Department of Ophthalmology, Alava University Hospital, Vitoria, Spain; 11 Las Claras Clinic, Salamanca, Spain; 12 Department of Ophthalmology, La Mancha Centro General Hospital, Alcázar de San Juan, Ciudad Real, Spain; 13 Department of Ophthalmology, Leon University Hospital, León, Spain

**Keywords:** Eye diseases, Paediatric research

## Abstract

To evaluate the age-related efficacy and safety of atropine 0.01% eye drops over 2 years for myopia control in a multicentric pediatric Spanish cohort. A non-controlled, interventional, prospective multicenter study was conducted as an extension of the Spanish Group of Atropine Treatment for Myopia Control Study (GTAM 1). Children aged 6–14 years with myopia from − 2.00 to − 6.00 D, astigmatism < 1.50 D and documented annual myopic progression of at least − 0.50 D under cycloplegic examination were recruited. From the original cohort of 105 participants, 92 children who had been receiving atropine 0.01% eye drops once nightly in each eye for 1 year continued their participation in this extended study (GTAM 2). All the patients underwent a standardized quarterly follow-up protocol, which included measurements of best-corrected visual acuity (BCVA), cycloplegic autorefraction, axial length (AL), anterior chamber depth (ACD), and pupil diameter. The study sample was divided into three age groups: 6–8, 9–11, and 12–14 years old. The mean change in cycloplegic spherical equivalent (SE) and axial length (AL) during the 24 months of follow-up was analyzed. Correlations between SE and AL, as well as the distribution of annual progression, were evaluated. Adverse effects were recorded using a specific questionnaire. Finally, 81 children completed the follow-up and were included in the analysis. Over the 2-year period, the mean changes in SE and AL were − 0.88 ± 0.60 D and 0.49 ± 0.25 mm, respectively. Additionally, 51 patients (63%) experienced SE annual progression lower than − 0.50 D. The correlation between the progression of SE and AL during the total period of treatment was mild (r = − 0.36; p < 0.001). There were no differences between the first and the second year of treatment in the progression of SE (− 0.42 ± 0.41 D versus − 0.45 ± 0.39 D; p = 0.69) or AL (0.25 ± 0.16 mm versus 0.23 ± 0.14 mm; p = 0.43). Older patients (12–14 years old) showed less AL progression than younger children (6–8 years old): 0.36 ± 0.18 mm versus 0.59 ± 0.30 mm; p = 0.01. Adverse effects were mild, infrequent, and decreased over time. On average, the myopia progression in control groups from other published biannual studies exceeded that observed in our study. Over 2 years, atropine 0.01% demonstrated a safe treatment for controlling myopia progression in a multicentric cohort of Spanish children. The effect remained stable during this period. Older patients exhibited a more favorable response in terms of AL enlargement. However, further studies are needed to investigate the age-related effect of low-dose atropine in the Caucasian population.

## Introduction

The increasing prevalence of myopia and its progression have become a public health and economic burden challenge worldwide^1^. The concern is greater in Asian countries due to a higher prevalence of myopia. However, this issue has also reached Western countries where myopia is increasing, presenting at an earlier age, and has been reported between 20 and 40% in adult populations^2,3^. The prevalence of childhood myopia is also on the rise among individuals of White ethnicity and European descent^4^*.*

The increasing concern regarding the rise in myopia prevalence stems from recognizing myopia as a progressive condition that significantly increases the risk of developing a range of ocular disorders, such as cataract, glaucoma, and retinal detachment. Besides, in high myopia, conditions like myopic maculopathy and optic neuropathy can result in substantial and irreversible visual impairment and blindness^5^.

Over the past decade, scientific evidence from rigorous clinical trials has indicated the potential of effective therapeutic options to slow down the progression of childhood myopia. As a result, treatments for myopia control are becoming more widely integrated into routine clinical practice worldwide^6^. Primary strategies aimed at preventing the onset and slowing down the progression of myopia encompass measures as the following: lifestyle interventions involving the optimization of environmental factors; pharmacological treatment utilizing topical atropine eye drops; and employment of optical devices such as multifocal spectacles, multifocal contact lenses with aspheric or discrete dual-focus designs, and orthokeratology^5^*.* Currently, there is a lack of universally adopted protocols for managing young myopic patients, despite the existence of multiple published position papers on the subject. Additionally, the availability and accessibility of different myopia control methods vary depending on geographical location^6^. In any case, controversy on the degree of evidence still exits on whether incremental benefits of these treatments are found over the years and whether the effects are sustained^7^*.*

Among the various pharmacological treatments, atropine has emerged as the most extensively studied and widely used antimuscarinic agent for managing myopia. Up to now, low concentration atropine is considered one of the most effective treatments for childhood myopia control in some studies^8,9^. The “Atropine for the Treatment of Myopia” (ATOM) clinical trials conducted in Singapore provided valuable insights into the effects of atropine on myopia progression. The initial phase of the ATOM 1 study demonstrated that the use of 1% atropine eye drops effectively controlled myopic progression over a two-year period (− 1.20 ± 0.69 D in placebo group versus − 0.28 ± 0.92 D in atropine 1% group), albeit with visual side effects that posed challenges to treatment compliance^10^. In the subsequent ATOM 2 study, lower doses of atropine (0.5%, 0.1%, 0.01%) were investigated and patients treated with 0.01% atropine experienced a favorable response with less rebound phenomenon (2-year progression: − 0.49 ± 0.63 D versus − 1.20 ± 0.69 D in placebo group)^11^. Subsequently, the “Low-Concentration Atropine for Myopia Progression” (LAMP) study conducted in Hong Kong, China, examined the efficacy and safety of low-concentration atropine eye drops (0.05%, 0.025%, and 0.01%) compared to a placebo. The study found that the effectiveness of the treatment was concentration dependent. At 2 years atropine 0.05% yielded the best response: − 0.55 ± 0.86 D progression versus − 1.12 ± 0.85 D with atropine 0.01%^12^. Although well tolerated in Asian children, 0.05% atropine has stronger and more significant side effects compared to 0.01% atropine in young Caucasian children, potentially compromising acceptance and compliance in this ethnicity. Impaired vision or reading difficulties were observed in 63.0% of Caucasian children treated with 0.05% atropine eye drops^13^.

While the majority of initial clinical trials and studies investigating atropine for myopia management focused on Asian populations, several studies with atropine conducted in non-Asian children have also demonstrated variable efficacy^14^. As a result, there is an increasing adoption of Asian clinical trial protocols by ophthalmologists in Western countries. Among these protocols, the most commonly utilized atropine dose is 0.01%^15,16^.

In 2017, we designed both a retrospective and an interventional prospective study on atropine 0.01% efficacy and safety among progressive myopic Spanish children. In the first year (phase 1) results of the Multicenter Group of Atropine Treatment for Myopia Control (GTAM) study, atropine was found to be effective and safe for myopia progression control in our multicenter children cohort. After 1-year of atropine 0.01% treatment, the mean SE progression was lower than the year before treatment (− 0.44 ± 0.41 D versus − 1.01 ± 0.38 D; p < 0.0001)^17^. On the other hand, both the ATOM-2 clinical trial and the LAMP study found that 0.01% atropine efficacy improved during the second year of treatment^12,18^. Besides, an age-dependent factor has also been reported with different response to atropine eye drops between older and younger children parallel to the greater inherent risk of myopia progression in the youngest children^19^. In fact, the LAMP study found that younger age was associated with a poorer response to the 0.01% atropine treatment compared to the 0.05% concentration^20^.

Our purpose was to investigate the 2-year effectiveness, the age-related response and the atropine 0.01% treatment side effects in a multicentric pediatric Spanish cohort. This extended study reports the phase 2 results of the multicenter Group of Atropine Treatment for Myopia Control (GTAM).

## Patients and methods

Methods and design details of the multicenter retrospective and interventional prospective study (GTAM study phase 1) have already been published^17^ and are briefly described here. Spanish children aged 6–14 years wearing single vision glasses to correct refractive error from − 2.00 to − 6.00 D, astigmatism < 1.50 D and documented myopic progression of at least − 0.50 D under cycloplegic examination over the previous year, were recruited and followed for two years from baseline visit. Patients were excluded from the study if they had ocular or systemic conditions affecting vision or refractive error, contraindications to atropine use, a history of amblyopia or strabismus, were undergoing myopia control treatments (e.g., prior atropine or pirenzepine use, use of any type of myopia control lenses), or had circumstances that could impede adherence to the study protocol. Participants who refused to discontinue the use of single vision contact lenses throughout the study were also excluded. Patients were recruited from October 2017 to April 2019 in 12 Spanish centers: POVISA Hospital, Vigo; Vigo University Hospital Complex; Lugo University Hospital; Ourense University Hospital Complex; Lorente Clinic (Ourense); A Coruña University Hospital Complex; Victoria de Rojas Ophthalmologic Institute (A Coruña); Navarra University Clinic; Alava University Hospital; Las Claras Clinic (Salamanca); Leon University Assistance Complex; and La Mancha Centro General Hospital.

All included patients were treated with 0.01% atropine sulfate eyedrops nightly in each eye for 24 months. Eyedrops were compounded and dispensed in Spanish Agency of Medicines and Medical Devices (AEMPS) authorized pharmacies in accordance with an identical procedure. The 0.01% atropine ophthalmic solution was prepared in a sterile manner (Atropine Sulfate 1 mg, Sodium Chloride ClNa 0.9%, Glacial Acetic Acid q.s., Sodium Acetate q.s. to pH = 5.0–6.0; Active Pharmaceutical Ingredient 10 ml), and was stored in Low Density Poliethylene multi-dose bottles.

Neither randomization nor placebo nor control group were established. The study was classified by the AEMPS as an observational prospective post-authorization study (*Classification Code: IPF-ATR-2017-01 on October 26, 2017*)*.* The study was approved by each of the referral Ethics Committee of all the participating centers. Written informed consent was obtained from parents or legal tutors of all participants. All procedures were conducted according to the Tenets of the Declaration of Helsinki.

Uniform study and exploration protocols were adhered to across all participating centers. The patients underwent a standardized follow-up protocol, which included an initial visit followed by a telephone consultation after two weeks to assess treatment tolerance and compliance. Subsequently, office controls were conducted at 4, 8, and 12 months (phase 1), and additional visits were scheduled at 16, 20, and 24 months (phase 2).

On each visit, best-corrected distance and near visual acuity were assessed using log-MAR charts, i.e., Early Treatment Diabetic Retinopathy Study (ETDRS) charts or equivalent. Ocular AL and ACD were measured on an IOL Master® (Carl Zeiss Meditec, Inc, Dublin, CA) available at each center, with an average of 6 measurements performed. Automatized measures of pupil diameter (IOL Master, Zeiss) were made with dim ambient light conditions. Cycloplegic autorefraction was performed at least 30 min after the third 1% cyclopentolate eyedrop and 3–5 readings of the spherical and cylinder components that had to differ less than < 0.25 D were obtained. SE was calculated as spherical power plus half of the cylinder power.

Compliance and treatment side effects were assessed through telephone interviews with parents two weeks after the baseline visit, and subsequently during office visits with both parents and children. Compliance was considered satisfactory if fewer than 5 days per month of treatment application were missed. A questionnaire was developed to record local side effects, such as ocular discomfort, light intolerance, and blurred near vision. These effects were quantified as mild, moderate, or severe based on symptom intensity and duration on the following day: mild (lasting less than 2 h), moderate (not impending ordinary activities but lasting more than 2 h), and severe (impeding ordinary activities for more than 3 h). Systemic adverse effects were documented as either present or absent, and if present, further analysis was conducted to determine the type of systemic pathology and its potential relationship with the use of atropine.

The main outcome of this study was myopia progression in terms of SE and AL changes over two years (combined phase 1 and phase 2). The secondary outcomes included SE and AL changes during the second year, annual SE progression distribution, the analysis of the age as a progression factor and side effects detection. In order to assess baseline age as an atropine treatment response influential factor, we divided the cohort in three different age groups (6–8, 9–11 and 12–14 years old, respectively) and analyzed possible differences in SE and AL progression over the two years of treatment.

### Statistical analysis

Sample size calculation has been described in detail^17^ and is briefly described here. The population of children in Spain aged 6–14 was reported to be 4,380,101 individuals in January 2017, according to the Spanish Statistical Office and the National Statistics Institute. Assuming a myopia prevalence of 6.7% among 10-year-old children of white ethnicity, our estimated number of myopic children in the Spanish reference population would be 293,467. To ensure a 95% confidence level with a 5% margin of error, we calculated a required sample size of 384 individuals. To account for potential dropouts, we set our sample size target at 400 participants, allowing for a 5% attrition rate.

Correlation analysis was performed with data from both eyes that permitted a pooled analysis of those values as a unique data for each patient. A descriptive analysis of the variables was also performed. Changes in the evaluated parameters (SE, AL, ACD, pupil diameter, BCVA at distance and near) were considered as the difference between the baseline data and the data at the end of the follow-up period. Lilliefors test was used to prove the statistical assumption of normality whereas t Student and Wilcoxon test were used to prove paired samples. Pearson correlation was used to analyze the relation between the SE and AL progression. To analyze if there were differences in SE and AL progression between the first and the second year of treatment Wilcoxon test was used after checking the data sets normality and normal distribution of variances using the Shapiro–Wilk and Snedecor F-tests. Anova test with Holm correction and Tukey HDS test were used to demonstrate differences in the progression of SE and AL between the three age-related groups after checking the data sets normality and normal distribution of variances using the Shapiro–Wilk and Bartlett tests.

The number of successful outcomes was measured using defined criteria^11,17^: success was achieved when there was an annual progression lower than − 0.50 D. Cases with a myopic progression falling between − 0.50 and − 0.99 D were considered to have moderate progression. Cases with an annual progression equal to or higher than − 1.00 D were categorized as showing severe progression or being non-responder patients. The annual percentage distribution of successful outcomes was also calculated.

The software used for the analysis was: R (4.0.3 version), RStudio (1.1.463 version) and IBM SPSS Statistics 20. p value < 0.05 was considered statistically significant.

## Results

### Patients

Out of the initial cohort of 105 participants, that received atropine 0.01% once daily in GTAM phase 1 study, 92 continued this extended phase 2 study. During the second year the situation that arose from the Covid-19 pandemic in Spain made it especially difficult to fulfill with the follow-up visits protocol; specifically, 10 patients did not fulfill the 20th month visit but continued enrolled in the study. Finally, 81 children reached the 24th month visit and were analyzed. Along the 2 years, 24 patients were excluded from the study (77% follow-up): 19 did not fulfilled the scheduled visits, 1 decided to wear orthokeratology contact lenses, and 4 presented an adverse event.

Demographic data is summarized in Table 1. Mean age was 9.74 ± 1.90 years (6–14 years), mean SE progression in the previous year was − 1.01 ± 0.38 D, 42 (51.85%) were female, 79 (97.53%) were Caucasian and 63 (77.78%) had brown colored iris.

**Table 1:** Demographic variables distribution.

Variable	n
Patients	81
Mean age ± SD (range), years	9.74 ± 1.90 (6–14)
SE Mean progression previous year, D	− 1.01 ± 0.38 D
Gender (female), N (%)	42 (51.85)
Race, N (%)	
Caucasian	79 (97.53)
Asian	2 (2.47)
Iris color (pigmentation), N(%)	
Dark	63 (77.78)
Medium	13 (16.05)
Light	4 (4.94)

### Ophthalmologic parameters

At the 24th month visit, mean increase in SE was − 0.88 ± 0.60 D and in AL 0.49 ± 0.25 mm. Table 2 shows the changes in the ophthalmologic parameters over the two year of the study. All parameters underwent significantly changes except for the distance and near best corrected visual acuity.

**Table 2:** Change in ophthalmic parameters over 2-year atropine 0.01% treatment.

Variable	First year atropine 0.01% treatment (n = 81)			
	Baseline visit	12 months	Mean change	p-value
SE (mean ± SD, D)	− 3.57 ± 1.12	− 4.00 ± 1.14	− 0.42 ± 0.41	< 0.001
AL (mean ± SD, mm)	24.56 ± 0.75	24.82 ± 0.77	0.25 ± 0.16	< 0.001
AC (mean ± SD, mm)	3.79 ± 0.33	3.82 ± 0.30	0.02 ± 0.26	0.019
Pupil size (mean ± SD, mm)	5.63 ± 1.38	6.28 ± 1.09	0.66 ± 1.08	< 0.001
Near VA (logMAR)	0.00 ± 0.02	0.00 ± 0.01	− 0.00 ± 0.02	0.853
Distance VA (logMAR)	0.00 ± 0.03	− 0.00 ± 0.04	− 0.01 ± 0.05	0.545

	Second year atropine 0.01% treatment (n = 81)			
	12 months	24 months	Mean change	p-value
SE (mean ± SD, D)	− 4.00 ± 1.14	− 4.43 ± 1.23	− 0.45 ± 0.39	< 0.001
AL (mean ± SD, mm)	24.56 ± 0.75	25.04 ± 0.79	0.23 ± 0.14	< 0.001
AC (mean ± SD, mm)	3.82 ± 0.30	3.85 ± 0.26	0.02 ± 0.20	0.031
Pupil size (mean ± SD, mm)	6.28 ± 1.09	6.45 ± 0.99	0.14 ± 0.66	0.045
Near VA (logMAR)	0.00 ± 0.01	0.00 ± 0.02	− 0.00 ± 0.23	0.950
Distance VA (logMAR)	-0.00 ± 0.04	− 0.01 ± 0.03	− 0.00 ± 0.02	0.141

	2 years atropine 0.01% treatment (n = 81)			
	Baseline visit	24 months	Mean change	p-value
SE (mean ± SD, D)	− 3.57 ± 1.12	− 4.43 ± 1.23	− 0.88 ± 0.60	< 0.001
AL (mean ± SD, mm)	24.56 ± 0.75	25.04 ± 0.79	0.49 ± 0.25	< 0.001
AC (mean ± SD, mm)	3.79 ± 0.33	3.85 ± 0.26	0.04 ± 0.27	0.004
Pupil size (mean ± SD, mm)	5.63 ± 1.38	6.45 ± 0.99	0.79 ± 1.21	< 0.001
Near VA (logMAR)	0.00 ± 0.02	0.00 ± 0.02	− 0.00 ± 0.03	1.000
Distance VA (logMAR)	0.00 ± 0.03	− 0.01 ± 0.03	− 0.01 ± 0.04	0.010

SE, spherical equivalent; SD, standard deviation; D, diopter; AL, Axial length; AC, anterior chamber; VA, visual acuity; logMAR, logarithm of minimun angle resolution.

**Figure 1:**
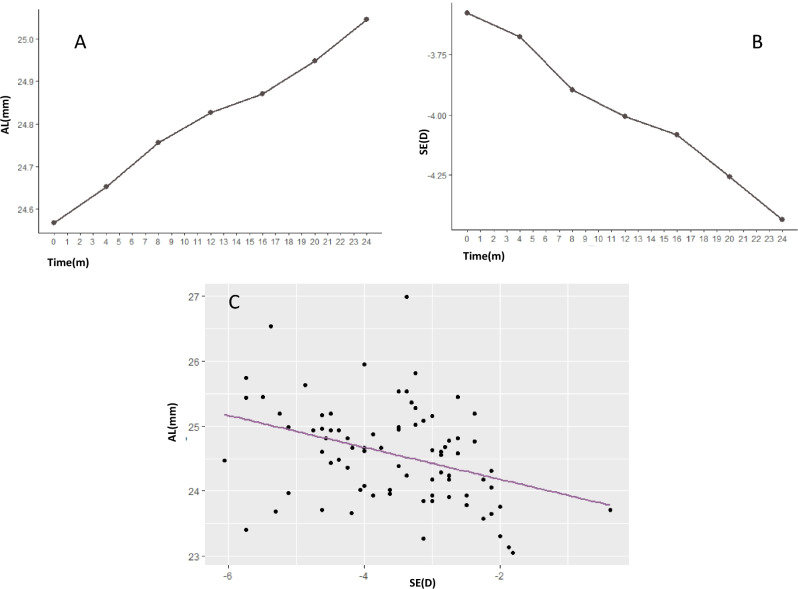
(**A**) Change in AL across the time. (**B**) Change in SE across the time. (**C**) AL and SE progression correlation (Pearson Correlation r: − 0.36; p < 0.001).

There were not differences between the first and the second year of treatment in progression of SE (− 0.42 ± 0.41 D versus − 0.45 ± 0.39 D; p = 0.69) or AL (0.25 ± 0.16 mm versus 0.23 ± 0.14; p = 0.43). The correlation between the progression of SE and AL during the total period of treatment was mild (r: − 0.36; p < 0.001) (Fig. 1).

**Figure 2:**
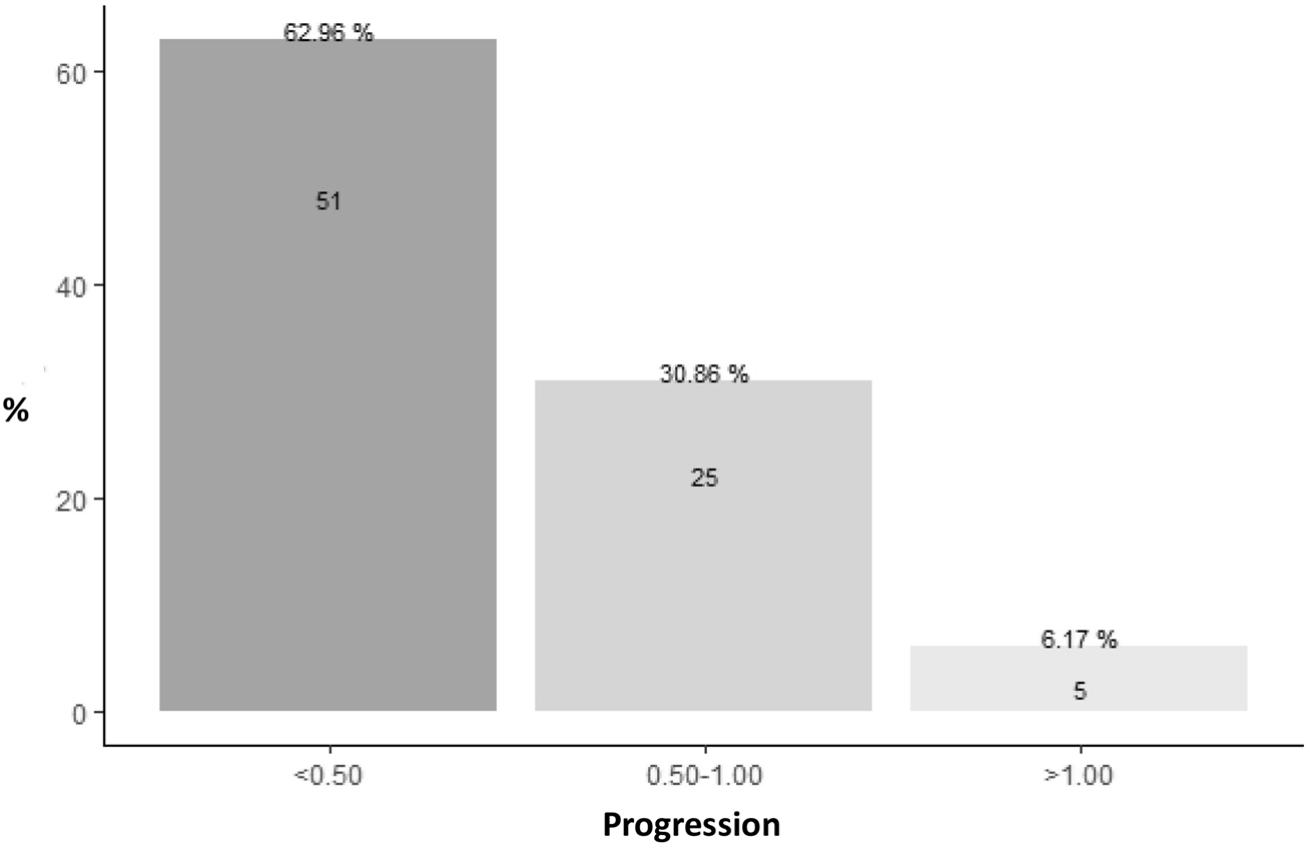
Distribution of SE annual progression during the 2-year treatment period.

Over the two years, 51 (62.96%) patients underwent an annual SE progression < − 0.50 D, and 6.17% were non-responders (SE annual progression > 1 D) (Fig. 2).

### Age-related progression

There were no differences in the progression of SE and AL between the first and second year of treatment for any of the age-related groups. We found no significant differences between the different age groups in the SE progression (p = 0.35), however we did find less AL progression in the oldest age group (12–14 years-old) compared with the youngest (6–8 years-old) (0.36 ± 0.18 mm versus 0.59 ± 0.30 mm; p = 0.01) (Tables 3 and 4).

**Table 3:** Change in SE and AL progression between the first and second year and over 2-year atropine 0.01% treatment according to age.

Age group, years (N)	0–12 months	12–24 months	0–24 months	p-value*
	SE (mean change ± SD, D)			
6–8 (23)	− 0.44 ± 0.52	− 0.47 ± 0.40	− 0.91 ± 0.75	0.648
9–11 (41)	− 0.43 ± 0.36	− 0.48 ± 0.35	− 0.91 ± 0.53	
12–14 (17)	− 0.41 ± 0.36	− 0.34 ± 0.47	− 0.75 ± 0.56	
	AL (mean change ± SD, mm)			
6–8 (23)	0.31 ± 0.21	0.27 ± 0.16	0.59 ± 0.30	0.021
9–11 (41)	0.25 ± 0.15	0.24 ± 0.13	0.49 ± 0.23	
12–14 (17)	0.19 ± 0.10	0.17 ± 0.13	0.36 ± 0.18	

N, patients; SE, spherical equivalent; SD, standard deviation; D, diopters; AL, axial length; SD, standard deviation.

p value*: ANOVA test.

**Table 4:** Change in AL progression at 24 months between age groups.

Age group (years)	Holm correction	Tukey HDS test
	p-value	p-value
1(6–8) *vs* 2 (9–11)	0.170	0.270
1 (6–8) *vs* 3 (12–14)	0.017	0.015
2 (9–11) *vs* 3 (12–14)	0.170	0.190

(Post hoc Anova tests).

### Adverse effects

Most of the observed side effects were mild. At 12 months: 1 (1.23%) patient had mild ocular discomfort, 2 (2.47%) patients had mild light intolerance and there were 2 patients with near vision difficulty, one (1.23%) was mild and the other (1.23%) was moderate. At 24 months only one (1.23%) patient had mild near vision difficulty. At the end of the study, only 4 of the 105 initially enrolled children had to be excluded due to adverse events: 2 of them were excluded due to nonspecific ocular discomfort, and the other 2 for experiencing mild concurrent systemic symptoms of tachycardia and vertigo. In both cases, symptoms were transient, ceased with treatment suspension, and did not require any other intervention. Although no specific investigation was performed, a causal relationship with the treatment is unlikely.

## Discussion

In this report we present the second-year results (phase 2) of the multicenter study of atropine 0.01% for myopia control (GTAM) in a Spanish cohort of children. Inspired by Asian clinical trials on atropine treatment for managing myopia progression, this study aimed to provide real-world evidence that can be applied to daily clinical practice in Spain. The study involved 12 participating centers spanning a large geographical area within the country. A standardized protocol was devised not only for conducting examinations and collecting data, but also for identifying potential treatment side effects through telephone surveys and office visits.

After a 2-year treatment with atropine 0.01% in our multicenter study, the mean increase in SE was − 0.88 ± 0.60 D, and in AL was 0.49 ± 0.25 mm. Comparing this to the year before treatment initiation, during which our cohort had progressed by − 1.01 ± 0.38 D in just 1 year, suggests a substantial reduction in myopia progression. Additionally, in our series, 63% of patients experienced an annual progression lower than − 0.50 D during the 2-year follow-up period.

In any case and considering that we had no control group for comparison, we can refer to other published biannual myopia progression studies that utilized atropine 0.01% and had similar baseline characteristics (Tables 5 and 6).

**Table 5:** Studies in Asian countries with atropine 0.01% and 2-years follow-up.

Author, year	Country, n (C:Control/A: Atropine)	Study design	Age (years) (range, mean, SD)			Baseline SE (D) (range, mean, SD/IC95)			Baseline AL (mm) (mean, SD/IC95)		Follow-up time (months)	Change of SE (D) (mean)		Control rate of SE progression (dif D, %)	Change of AL (mm) (mean)*		Control rate of AL progression (dif mm, %)
			Range	Control	Atropine 0.01%	Range	Control	Atropine 0.01%	Control	Atropine 0.01%		Control	Atropine 0.01%		Control	Atropine 0.01%	
Chia, 2012 ATOM 2	Singapore (C: 200*/A: 84)	RCT	6–12	9.2	9.5	≤ − 2.0	− 3.58 (1.2)	− 4.5 D (1.5)	24.80 (0.8)	25.1 (1.0)	0–12	− 0.76*	− 0.43	0.33 (43.4)	0.20*	0.24	− 0.04
											12–24	− 0.44*	− 0.06	0.38 (86.4)	0.18*	0.17	0.01
											0–24	− 1.20*	− 0.49	0.71 (59.2)	0.38*	0.41	− 0.03
Yam, 2020 LAMP 2	Hong Kong, China (C: 111/A: 110)	RCT	4–12	8.42 (1.72)	8.35 (1.8)	≤ − 1.0	− 3.85 (1.95)	− 3.99 (1.94)	24.82 (0.97)	24.78 (1.02)	0–12	− 0.81	− 0.64	0.17 (21.0)	0.41	0.35	0.06 (14.6)
											12–24	− 0.69	− 0.48	0.21 (30.4)	0.35	0.25	0.10 (28.6)
											0–24	– 1.58	− 1.12	0.46 (29.1)	0.76	0.59	0.17 (22.4)
Cui, 2021	Mainland China (C: 120/A:142)	RCT	6–14	9.3 (1.4)	9.4 (1.7)	− 1.25 to − 6.00	− 2.66 (− 2.70, − 2.62)	− 2.76 (− 2.81, − 2.71)	24.54 (24.4, 24.7)	24.60 (24.5, 24.7)	0 − 12	− 0.70	− 0.47	0.23 (32.9)	0.46	0.37	0.09 (19.6)
											12 − 24	− 0.63	− 0.46	0.17 (27.0)	0.42	0.35	0.07 (16.7)
											0 − 24	− 1.33	− 0.93	0.40 (30.1)	0.88	0.72	0.16 (18.2)
Hieda, 2021 ATOM J	Japan (C: 86/A: 85)	RCT	6–12	8.98 (1.50)	8.99 (1.44)	− 1.00 to − 6.00	− 2.98 (− 3.27, − 2.69)	− 2.91 (− 3.20, − 2.62)	24.51 (24.3, 24.7)	24.43 (24.3, 24.6)	0–12	− 0.77	− 0.69	0.08 (10)	0.39	0.35	0.04 (10.2)
											12–24	− 0.77	− 0.57	0.20 (25.9)	0.38	0.28	0.10 (26.3)
											0–24	− 1.48	− 1.26	0.22 (14.8)	0.77	0.63	0.14 (18.2)

*Control Group from ATOM1.

**Table 6:** Studies in non-Asian countries with atropine 0.01% and 2-years follow-up.

Author, year	Country, n (C:Control/A: Atropine)	Study design	Age (years) (range, mean, SD)			Baseline SE (D) (range, mean, SD/IC95)			Baseline AL (mm) (mean, SD/IC95)		Follow-up time (months)	Change of SE (D) (mean)		Control rate of SE progression (dif D, %)	Change of AL (mm) (mean)*		Control rate of AL progression (dif mm, %)
			Range	Control	Atropine 0.01%	Range	Control	Atropine 0.01%	Control	Atropine 0.01%		Control	Atropine 0.01%		Control	Atropine 0.01%	
Larkin, 2019	USA (C:98/A:100)	Retro	6–15	9	9	− 0.25 to − 8.00	− 2.8 (1.6)	− 2.8 (1.6)			0–12	− 0.6	− 0.2	0.40 (66.7)			
											12–24	− 0.6	− 0.1	0.50 (83.3)			
											0–24	− 1.2	− 0.3	0.90 (75)			
Moriche-Carretero, 2021	Spain (C: 168/A:171)	RCT	5–11	7.24 (1.77)	7.37 (1.54)	− 1.25 to − 4.25	− 2.16 (0.62)	− 2.13	24.26 (0.91)	24.22 (0.66)	0–24	− 0.76	− 0.51	0.25 (32.8)	0.37	0.20	0.17 (45.9)
Lee S, 2022	Australia (C: 49/A104)	RCT	6–16	12.2 (2.5)	11.2 (2.7)	≤ − 1.50	− 3.56 (− 4.56, − 2.75)	− 3.13 (− 4.08, − 2.48)	24.7 (24.4, 25.4)	24.6 (24.2, 25.2)	0–12	− 0.53	− 0.31	0.22 (41.5)	0.25	0.16	0.09 (36)
											12–24	− 0.25	− 0.33		0.62	0.36	0.26 (41.9)
											0–24	− 0.78	− 0.64	0.14 (18.9)	0.38	0.34	0.04 (10.5)
Zadnik K, 2023 CHAMP	USA, UK, Spain, Hungary, Netherlands, Ireland (C: 165/A: 164)	RCT, Multic	3–17			− 0.50 to − 6.00	− 3.72 (1.42)	− 3.41 (1.49)	24.33 (0.84)	24.37 (0.81)	0–12	− 0.57	− 0.45	0.12 (21)	0.37	0.30	0.07 (18.9)
											12–24	− 0.43	− 0.34	0.09 (20.9)	0.26	0.21	0.05 (19.2)
											0–24	− 1	− 0.79	0.21 (21)	0.63	0.51	0.12 (19)
Perez Flores, 2023 GTAM	Spain (A: 105)	Prospec, Multic	6–14		9.74 (1.90)	− 2.00 to − 6.00		− 3.57 (1.12)		24.56 (0.75)	0–12		− 0.42			0.25	
											12–24		− 0.45			0.24	
											0–24		− 0.88			0.49	

Retro, retrospective study; RCT, randomized clinical trial; Multic, multicenter study; Prospec, prospective study.

Among the Asian studies, the Singapore study ATOM 1^11^ found mean SE progression of − 1.2 D and AL increases of 0.38 mm after 2 years in eyes treated with placebo versus − 0.49 D and 0.41 mm in those treated with atropine 0.01%. In a clinical trial conducted in Mainland China the placebo group showed a SE progression of − 1.33 and AL increase 0.88 mm at 2-years versus − 0.93 D and 0.72 mm in the atropine 0.01% group^21^. The LAMP 2 study in Hong Kong found an estimated 2-year SE progression in the placebo group of − 1.58 D and AL increase of 0.76 mm versus − 1.12 D and 0.59 mm respectively in the atropine 0.01% group^12,22^. In a clinical trial in Japan (ATOM J) the 2-year SE progression in the placebo group was − 1.48 D and 0.77 mm of AL increase versus − 1.26 D and 0.63 in the atropine 0.01% group^23^.

Regarding non-Asian studies, Larkin in the USA found a mean SE progression of − 1.2 D at 2-years follow up in the placebo group versus − 0.3 D in the atropine 0.01% group^24^. The CHAMP phase 3 study, a multicenter multiethnic trial with a 74% of non-Asian participants, showed a 2-year SE progression in the placebo arm of − 1.00 D and an AL increase of 0.63 mm, versus -0.79D and 0.51 mm, respectively, in the atropine 0.01% group^25^*.*

In summary, most of these studies showed that the control group presented a 2-year SE progression ≥ -1D and AL progression ≥ 0.60 mm. In fact, among the corresponding eight control groups listed in Tables 5 and 6, the mean 2-year SE and AL progression were − 1.17 ± 0.30 D and 0.59 ± 0.22 mm, respectively. Therefore, on average, the progression in these control groups was greater than in our study.

On the other hand, a lower rate of progression over 2 years has been observed among the untreated participants of a Spanish series, with a mean SE progression of − 0.76 D and an AL increase of 0.37 mm, versus − 0.51 D and 0.20 mm, respectively, in the atropine 0.01% treated group^26^. Interestingly, in another recent publication with Spanish children, the 5-year progression in the no-treatment group was − 0.92 ± 0.56 D, with an AL increase of 0.49 ± 0.34 mm^27^. These findings highlight the necessity to compare studies not only with similar age and ethnicity but also with similar baseline characteristics: the proportion of mild myopic patients in these two series seem to be higher than ours: a mean SE of − 2.14 D (range − 0.50 to − 4.50 D) is far from our − 3.57 D (range from − 2 to – 6 D). We designed a study that could be comparable with Asian randomized clinical trials and therefore we only included children with at least – 2 D of myopia and demonstrated progression in the previous year. Notably, a significant disparity in baseline SE was observed when reviewing the baseline characteristics among the studies (Table 5). For instance, the ATOM 2 and LAMP 2 studies reported notably higher myopia averages (− 4.5 D and − 3.77 D in the 0.01% atropine group, respectively) compared to a Spanish series^26^. While controversy surrounds baseline myopia as a progression factor, these differences could potentially influence the results, given that progression seems to occur faster in cases of moderate myopia compared to mild myopia^19,28^. Consequently, when evaluating the efficacy of 0.01% atropine treatment in children, it is vital to approach the comparison of these outcomes with caution, considering the diverse study designs and the baseline characteristics of the participants involved^29^.

The optimal treatment strategy of atropine treatment at low concentration in terms of both, dose and duration of treatment, is not yet well established^30,31^. Although atropine 0.01% is currently the most generally used concentration in clinical trials for childhood myopia intervention, there had been contradictory results in the literature due to the characteristics of the responders to this treatment^29^. Even though the initial clinical trials demonstrated a clear treatment efficacy with atropine 0.01%^18^, subsequent studies have reported a less favorable response^26,32,33^. As emphasized, variations in population, study design, and inclusion criteria may have influenced these differing outcomes. Based on the studies listed in Table 5, the effectiveness of treatment, in terms of differences in 2-year SE progression, ranged from 0.14 to 0.9 D less in the atropine 0.01% groups compared to the control groups.

Commonly adopted protocols are based on the results of Asian clinical trials; however, disparities in results have been already evidenced between Asian and non-Asian populations^26,34^. It is not clear that ethnicity per se determines the response to atropine. The difference in treatment efficacy between Asian and Western children may depend not only on genetic factors but also on environmental and sociocultural factors. These latter factors appear to play a major role in the onset and progression of myopia. Recently, a study on the effect of atropine 0.01% in a multiracial cohort in Australia^32^, found that atropine 0.01% was not effective in South and East Asian children and had only a moderate effect in European children. The authors acknowledged that due to their study design the benefits of 0.01% atropine may had been underestimated and that the sample sizes of the different ancestry groups were small and therefore, larger samples of multiracial participants were needed to confirm their findings^32^.

In our case, we found that atropine 0.01% had similar effect during the first and second year of treatment in terms of mean SE progression (− 0.42 ± 0.41 D and − 0.45 ± 0.39 D, respectively). However, these results differ from the results of the ATOM 2 (phase 2) and LAMP 2 Asian studies that found better efficacy with atropine 0.01% during the second year^12,35,36^. While some studies have found a decrease in treatment efficacy^37,38^, other have also found similar efficacy over the 2-years of treatment^21,33^.

In terms of age, we found that after 2 years of treatment there was less AL progression in the 12–14 years-old group than in the 6 to 8 years-old group (0.36 ± 0.18 mm versus 0.59 ± 0.30 mm). Although there were also differences in SE progression among different age groups (− 0.75 ± 0.56 D in the oldest group versus − 0.91 ± 0.75 in the youngest), they did not reach statistical significance. The small sample size of the age groups could have affected these results. AL is a more precise measurement, and it is likely easier to detect smaller statistically significant differences compared to SE. Our results differ from some studies that found better response in younger children^26,29^, and agree with other authors that have described less efficacy of atropine treatment in younger children^20,34,39–42^. Younger children may require higher concentrations to achieve similar reductions in myopic progression, and also a higher rebound effect has been described among them^43^. Younger age (generally younger than 10 years-old) has been significantly associated with faster myopia progression in a number of studies independently of ethnicity^40,44,45^. Tideman et al.^43^ described the longitudinal changes in AL in a European children cohort aged between 6 and 9 years old. Among them, the myopic children had an eye growth rate of 0.34 mm/year. The NICER study, also conducted on European children, found that AL progressed faster in the myopic younger cohort aged 6–16-years (0.30 mm/year) compared to the older 12–22-year cohort (0.15 mm/year)^43^. We also found a higher AL increase between the 6–8-year-old group (0.31 mm/year) compared to the 12–14-year-old group (0.19 mm/year) (Table 3). Logically, this age effect of atropine treatment could be related to the slowing of AL elongation with age. The annual slowing is considered to be an average of 15% per year both in Asian and non-Asians myopes^46^. On the other hand, this finding also highlights the need to establish different age groups when planning myopia intervention studies. In fact, among our comparative studies (Tables 5 and 6), there were significant age differences. Since the mean age of children included in our study was 9.7 years old, the children in the treatment group in the other Spanish series^26^ were 7.3 years old, and in the Australian series^32^, they were 11.2 years old. Asian series had a more similar age distribution to ours, although LAMP 2 study^12^ included children as young as 4 years old. Naturally, the estimated overall treatment effect could be affected by the range and distribution of ages in the cohort of children.

As far as we know, in addition to age, other factors that could modify the response to treatment have been analyzed with different results^40^. Interestingly, a recent study suggests that the retinal function at baseline could serve to identify the best responders to treatment with atropine 0.01%^47^.

Concerning the correlation of progression between SE and AL over the 2 years of treatment, we found it to be moderate. The anti-myopic effect of atropine as a treatment for myopia control is mainly due to reducing the AL, but there exist discrepancies regarding the effect of the treatment on the AL^21,26,33,34,48^. We know that the changes in SE and AL during growth are not exactly parallel and more studies with larger sample sizes are necessary to detect the difference in AL elongation between patients treated with atropine and controls^34,48^. In any case, AL data are more representative to determine the treatment effects of atropine since measurements are more accurate and not influenced by accommodative changes^33^.

Regarding the side effects of atropine 0.01% treatment, our developed questionnaire enabled us to quantify their severity. In agreement with other authors^11,19^ we found that the adverse effects were mostly mild and temporary and did not hinder treatment compliance. None of the patients required photochromatic protection or optical correction for near vision. We observed a mean increase of 0.79 mm in pupil diameter, which aligns with findings from Asian and non-Asian studies reporting a range between 0.5 and 1 mm^49,50^. At the initiation of the treatment, 15% of patients experienced mild ocular discomfort, 6.5% reported mild light intolerance, and 5.4% had mild difficulties with near vision. These side effects reduced to 1–2% at 12 months, and by the end of the 24-month period, only one patient (1.2%) experienced mild near vision difficulties. The second phase of our study confirms that the side effects of atropine 0.01% treatment are minimal, and there is a progressive decrease in their occurrence over time. Similar findings have been described in other studies involving the Caucasian population, where atropine 0.01% treatment has been well-tolerated^26,33^.

In this study, we encountered several limitations that should be acknowledged:

1. The lack of a placebo control group is a major drawback of our study design. Since our initial aim was to conduct a real-world study without financial support from companies, we faced difficulties in including a placebo group. Additionally, the growing success of therapies to slow myopia progression has raised ethical concerns about randomizing children to placebo or standard groups in clinical trials. Therefore, judging efficacy against an untreated control group may depend on comparison to historical, untreated control groups^6,40^, as we have done here. In addition, our study provides baseline data on the progression of the refractive error during the year prior to the start of atropine treatment.
2. The sample size was notably smaller than the ideal calculated sample size, a common challenge in Western studies due to the lower prevalence of myopia compared to Asia. Additionally, there was a significant increase in dropouts attributed to disruptions caused by the COVID-19 pandemic.
3. Family or environmental factors were not considered for the analysis.
4. In contrast to financially supported clinical trials, our study involved the compounding of the drug at multiple local sites, which may have introduced variations in concentrations and quality. While we did not conduct random sampling of the drug from different sites to test for concentration and stability, it is important to note that only authorized pharmacies approved by the Spanish Agency of Medicines were involved in compounding the drug. These pharmacies adhered to a strict, identical written protocol during the compounding process. However, it is worth mentioning that a recent study showed differences in formulation and inactive ingredients that could affect the efficacy and safety of atropine at low concentrations^51^.

On the other hand, having conducted a prospective multicentric study with 2 years-follow up period, with a similar mean age and baseline SE than the most important clinical trials; it makes feasible the comparison with other relevant studies. Diversity of study designs have conducted to some contradictory results assessing the characteristics of responders to 0.01% atropine^29^. Clarifying the associated factors behind poor responses to low-concentration atropine remains a significant challenge when making decisions about concentration adjustments or transitioning to alternative or combined therapies^20^.

## Conclusions

After 2 years of treatment, atropine 0.01% has proven to be safe for controlling myopia progression in Spanish children, with its effect remaining stable over time. The treatment appears to be more effective in older children. However, to implement a treatment onset protocol and determine the appropriate dosage, further studies are required to analyze the age-related effect of low-dose atropine in the Caucasian population.

## Data Availability

Correspondence and requests for materials should be addressed to J.B.B.
